# Racial Disparities and the Use of Artificial Intelligence for Predicting Maternal Mortality: A Literature Review

**DOI:** 10.3390/epidemiologia7030081

**Published:** 2026-06-10

**Authors:** Gustavo Gonçalves dos Santos, Anuli Njoku, Ana Carolina Pereira Mass, Ellen Eduarda Santos Ribeiro, Letícia Eduarda de Oliveira, Maria Julia Cunha Silva Lima, Taís de Abreu Ferro, Lilian Reinaldi Ribeiro Pirozi, Antônio Augusto de Freitas Peregrino, Célia Maria Pinheiro dos Santos, Lucia Helena Ferreira Viana, Marilda Gonçalves de Sousa, Carla Helena Cappello, Cely de Oliveira, Luis Henrique de Andrade, Cindy Ferreira Lima, Isabelle Cristinne Pinto Costa

**Affiliations:** 1 Programa de Pós-Graduação em Enfermagem (PPGENF/UnG), Universidade Guarulhos, Guarulhos 07023-070, SP, Brazil; 2 Escola de Enfermagem, Programa Nacional de Pós-Doutorado, Programa de Pós-Graduação em Enfermagem, Universidade Federal de Alfenas (UNIFAL-MG), Alfenas 37130-001, MG, Brazil; isabelle.costa@unifal-mg.edu.br; 3 Department of Public Health, College of Health and Human Services, Southern Connecticut State University (SCSU), New Haven, CT 06515, USA; njokua3@southernct.edu; 4 College of Health Sciences and Public Policy, Walden University, Minneapolis, MN 55401, USA; 5 Faculdade de Medicina de Marília, Residência Integrada Multiprofissional em Saúde (FAMEMA), Marília 17519-470, SP, Brazil; carolinapmass@gmail.com; 6 Centro de Ciências da Saúde, Departamento de Enfermagem, Programa de Pós-Graduação em Enfermagem, Universidade Federal do Piauí (UFPI), Teresina 64049-550, PI, Brazil; ellenribeirosr@gmail.com; 7 Pontifícia Universidade Católica de Minas Gerais (PUC/MG), Poços de Caldas 37714-620, MG, Brazil; leticia.eduarda.oliv@gmail.com; 8 Faculdade de Ciências Médicas da Santa Casa de São Paulo (FCMSCSP), São Paulo 01221-020, SP, Brazil; majucunha002@gmail.com; 9 Departamento Materno-Infantil e Psiquiátrica, Programa de Pós-Graduação em Enfermagem, Escola de Enfermagem da Universidade de São Paulo, (EEUSP), São Paulo 05403-000, SP, Brazil; tais.ferro@fcmsantacasasp.edu.br; 10 Escola de Enfermagem Alfredo Pinto, Programa de Pós-Graduação em Enfermagem e Biociências, Universidade Federal do Estado do Rio de Janeiro, (EEAP/PPGENFBIO/UNIRIO), Rio de Janeiro 22290-180, RJ, Brazil; antonio.peregrino@gmail.com; 11 Departamento de Ciências Radiológicas, Universidade do Estado do Rio de Janeiro (DCR/UERJ), Rio de Janeiro 20550-900, RJ, Brazil; lilianreinaldi@edu.unirio.br; 12 Faculdade de Medicina de Botucatu, Programa de Pós-Graduação em Saúde Coletiva, Universidade Estadual Paulista “Júlio de Mesquita Filho” (FMB/UNESP), Botucatu 18618-687, SP, Brazil; celiapinheiro5@gmail.com; 13 Centro Universitário Piaget (UNIPIAGET), Suzano 08673-270, SP, Brazil; luciaviana671@gmail.com; 14 Centro Universitário das Faculdades Metropolitanas Unidas (FMU), São Paulo 01503-001, SP, Brazil; marildagsouza@yahoo.com; 15 Universidade de Ribeirão Preto (UNAERP), Guarujá 11440-003, SP, Brazil; ccappello@unaerp.br; 16 Universidade Santa Cecília (UNISANTA), Santos 11045-907, SP, Brazil; celyoliveira@unisanta.br; 17 Centro Universitário Estácio, Santo André 09015-070, SP, Brazil; andrade.luis@estacio.br; 18 Organização Pan-Americana da Saúde (OPAS), Secretaria do Estado de Saúde de Minas Gerais, Minas Gerais 31630903, MG, Brazil; cindy.lima@alumni.usp.br

**Keywords:** artificial intelligence, hypertension, postpartum hemorrhage, maternal mortality, racial disparities in health

## Abstract

Background: Maternal mortality remains a major global health challenge, disproportionately affecting black and Indigenous women. Hypertensive disorders of pregnancy and postpartum hemorrhage are the leading direct causes of maternal death. Artificial intelligence (AI) tools have emerged as potential strategies for predicting these complications, yet concerns persist about their equity and validation across racial groups. Methods: A rapid review was conducted in five databases, PubMed, EMBASE, Web of Science, Scopus and LILACS, to synthesize recent evidence on the use of AI for preventing maternal mortality due to hypertension and postpartum hemorrhage. Studies published in the last five years that included racial or ethnic data were selected and analyzed narratively. Results: Ten studies met the inclusion criteria, showing high predictive accuracy of AI models (AUROC often >0.95) for severe maternal outcomes. However, few models incorporated racial variables or underwent external validation in racially diverse or low-resource populations. Evidence suggests that unrepresentative datasets may perpetuate or exacerbate existing health inequities. Conclusions: AI demonstrates strong technical performance in predicting maternal complications but limited equity in application. Broader racial representation, external validation, and ethical governance are essential for ensuring that AI-based tools reduce rather than reinforce racial disparities in maternal mortality.

## 1. Introduction

Maternal mortality remains a major and deeply unequal global public health problem. In 2023, an estimated 260,000 women died from causes related to pregnancy and childbirth, with more than 90% of these deaths occurring in low- and middle-income countries and largely preventable through timely and high-quality obstetric care. These figures highlight not only the magnitude of maternal mortality but also its strong association with social stratification and unequal access to health services, which disproportionately expose vulnerable populations to avoidable risks. Conceptually, maternal mortality results from the interaction between biological processes, health system performance, and structural determinants that shape exposure to risk and access to quality care [1].

Among the leading direct causes of maternal death worldwide are postpartum hemorrhage (PPH) and hypertensive disorders of pregnancy, particularly pre-eclampsia and eclampsia. Severe PPH, often associated with uterine atony or other obstetric complications amenable to rapid intervention, accounts for a substantial proportion of immediate maternal deaths. Hypertensive disorders, in turn, are associated with seizures, multiple organ failure, and a significant number of maternal deaths each year. Evidence indicates that late recognition of complications, shortages of essential supplies such as magnesium sulfate, and delays in definitive interventions are key contributors to these fatal outcomes [2,3].

Evidence-based interventions exist and can substantially reduce mortality from these conditions. Magnesium sulfate decreases the risk of progression from severe pre-eclampsia to eclampsia, while tranexamic acid and the active management of the third stage of labor reduce deaths from postpartum hemorrhage when administered promptly. However, the equitable implementation of these interventions remains limited by disparities in infrastructure, workforce training, and supply chains, particularly in settings with lower obstetric care capacity. Consequently, preventable maternal deaths reflect not only clinical failures but also broader systemic and structural deficiencies within health systems [3,4].

These disparities are also expressed along racial and ethnic lines. Studies indicate that hypertensive disorders of pregnancy and postpartum hemorrhage are disproportionately more frequent and severe among Black women. Analyses of large hospital databases show a higher prevalence of hospitalizations for PPH and a greater contribution of this complication to maternal deaths in this population, indicating a higher disease burden [5]. Research on social determinants further demonstrates that factors such as residential segregation, chronic psychosocial stress, and socioeconomic deprivation contribute to these disparities, highlighting the role of structural racism in shaping maternal risk. Epidemiological studies across different contexts consistently report increased risk of PPH among women from minority ethnic groups, particularly among Black women [6,7].

In addition to differences in the incidence of complications, inequities in clinical management have also been documented. Retrospective cohort studies show that Black women experiencing postpartum hemorrhage are less likely to receive higher-level therapeutic interventions or timely definitive procedures, suggesting that unequal treatment contributes directly to worse maternal outcomes [8]. Surveillance data and maternal death reviews similarly reveal higher concentrations of deaths from hemorrhage among Black, Indigenous, and socioeconomically vulnerable women, often associated with delays in recognition and response even among those who had access to prenatal care [9].

Within this context, artificial intelligence (AI) has emerged as a promising tool to support early identification of obstetric complications and improve maternal health outcomes. Predictive models for pre-eclampsia and postpartum hemorrhage, early warning systems based on vital sign monitoring, and algorithms capable of analyzing large volumes of clinical data have demonstrated high performance in retrospective studies and promising results when integrated into clinical workflows [10,11].

However, the introduction of AI into obstetric care also raises important concerns related to equity. Algorithms trained on unrepresentative datasets may reproduce or amplify existing racial inequalities, potentially underestimating risk in minority populations or generating inappropriate clinical recommendations. Research on algorithmic justice shows that biases may arise from skewed training datasets, the use of biased proxies, or the absence of performance evaluation stratified by race or skin color, leading to systematically worse outcomes for Black, Indigenous, and other marginalized groups [12,13].

Given this context, it is essential to critically analyze racial inequalities associated with the use of AI in maternal health. Therefore, this study aims to describe and analyze, through a rapid literature review, how artificial intelligence models applied to maternal health incorporate or neglect ethnic and racial inequalities, with particular attention to hypertension and postpartum hemorrhage, two major contributors to severe maternal morbidity and mortality. The analysis seeks to identify how limited representativeness and algorithmic bias may disproportionately affect Black women and other marginalized populations.

## 2. Materials and Methods

A rapid literature review was conducted with the aim of synthesizing evidence on the use of AI tools in preventing maternal mortality due to gestational hypertension and postpartum hemorrhage in black women. This rapid review followed systematic review principles but applied predefined methodological simplifications such as restrictions on publication period, languages, and scope of the search to enable the accelerated production of evidence, as recommended for rapid review methodologies [14]. The protocol for this review can be found on the Open Science Framework (OSF) platform (https://doi.org/10.17605/OSF.IO/B76MS).

The choice of rapid review methodology is justified by the highly dynamic and disruptive nature of the field of AI in healthcare. Unlike traditional clinical areas, where evidence is consolidated over decades, machine learning technologies and predictive models evolve in extremely short cycles. In this scenario, a conventional systematic review could result in conclusions that are already outdated at the time of publication. Therefore, rapid review was selected as the most appropriate tool to provide a timely and critical synthesis of emerging evidence, allowing managers and healthcare professionals to identify risks of algorithmic biases in real time, supporting strategic decisions on the implementation of technologies that directly impact equity in maternal health.

Unlike a conventional systematic review, this rapid review adopted strategic methodological accelerations to allow for a timely synthesis of evidence on AI and maternal mortality. These simplifications were based on Cochrane guidelines, focusing on maintaining scientific rigor despite the reduced timeframe. The main modifications compared to the Preferred Reporting Items for Systematic reviews and Meta-Analyses items are detailed below: Item 7 (Databases and Search Strategy): The search was restricted to the last five years and focused on five main databases to ensure that the analysis focused on peer-validated AI technologies ready for immediate clinical implementation; Item 8 (Study Selection): One reviewer was used for the initial screening of titles and abstracts, with a 20% sample verification by a second senior reviewer to ensure adherence to inclusion criteria. Single screening is a recognized speedup in rapid reviews to optimize turnaround time without substantial loss of sensitivity; Item 10 (Data Extraction): Data extraction was performed by a primary reviewer and checked by a second reviewer, rather than independent double extraction, to reduce workload and consensus time while maintaining accuracy through the checking step; Item 11 (Risk of Bias Assessment): The risk of bias assessment was simplified, focusing on the core domains that impact clinical prediction and algorithmic fairness, to prioritize the discussion of applicability and racial bias over an exhaustive psychometric analysis of all risk components.

The research question was constructed based on the PICO acronym [15], considering pregnant and postpartum women as population (P); the use of AI tools, predictive models or monitoring systems as exposure/intervention (I/E); standard care without AI or non-racialized populations as comparator (C); and maternal mortality due to gestational hypertension or postpartum hemorrhage as outcome (O).

Inclusion criteria were established that encompassed studies published in the last five years, in English, Portuguese or Spanish. In addition to studies that presented data stratified by race, studies that, while not providing specific quantitative analyses on race, explicitly discussed issues of racial inequality, algorithmic bias, or the need for validation of artificial intelligence in racialized populations were also included. These studies were considered relevant because they conceptually contributed to the understanding of the ethical, methodological, and equity implications of applying AI to maternal health. Thus, the review encompasses both empirical evidence and critical analyses that address the interface between AI and racial inequalities in the prevention of maternal mortality. Exclusion criteria included studies without racial distinction, without maternal and obstetric application of AI, single case reports, editorials or letters to the editor without empirical data, and works without access to the full text.

The search was conducted in the U.S. National Library of Medicine (PubMed)/Medical Literature Analysis and Retrieval System Online (MEDLINE), Excerpta Medica dataBASE (EMBASE), Web of Science, Scopus, and the Latin American and Literatura Latino-Americana e do Caribe em Ciências da Saúde (LILACS) databases. A combination of controlled vocabulary (MeSH/DeCS) and free-text terms related to maternal mortality, hypertensive disorders of pregnancy, postpartum hemorrhage, artificial intelligence, and racial or ethnic inequalities was used. Search strategies were adapted to the syntax and indexing standards of each information source, following the same conceptual structure.

Below, we present the example of the strategy applied: (“Maternal Mortality”[Mesh] OR “Maternal Mortality”[tiab] OR “maternal death”[tiab] OR “maternal deaths”[tiab]) AND (“Hypertension, Pregnancy-Induced”[Mesh] OR “Pre-Eclampsia”[Mesh] OR “Eclampsia”[Mesh] OR “Postpartum Hemorrhage”[Mesh] OR “hypertensive disorders of pregnancy”[tiab] OR “pregnancy-induced hypertension”[tiab] OR “gestational hypertension”[tiab] OR “pre-eclampsia”[tiab] OR “preeclampsia”[tiab] OR “eclampsia”[tiab] OR “postpartum hemorrhage”[tiab] OR “post-partum hemorrhage”[tiab]) AND (“Artificial Intelligence”[Mesh] OR “Machine Learning”[Mesh] OR “Algorithms”[Mesh] OR “Decision Support Systems, Clinical”[Mesh] OR “artificial intelligence”[tiab] OR “machine learning”[tiab] OR “predictive model”[tiab] OR “predictive models”[tiab] OR “risk stratification”[tiab] OR “clinical decision support”[tiab]) AND (“Ethnic Groups”[Mesh] OR “African Continental Ancestry Group”[Mesh] OR “Health Status Disparities”[Mesh] OR “racial disparities”[tiab] OR “ethnic disparities”[tiab] OR “racial inequality”[tiab] OR “ethnic inequality”[tiab] OR “Black women”[tiab] OR “women of African descent”[tiab]). The PubMed search was conducted using a combination of controlled terms (Medical Subject Headings—MeSH) and free terms (Title/Abstract). The complete PubMed search strategy was: ((((“Artificial Intelligence”[MeSH] OR “Machine Learning”[MeSH] OR “Algorithms”[MeSH])) AND ((“Maternal Mortality”[MeSH] OR “Postpartum Hemorrhage”[MeSH] OR “Hypertension, Pregnancy-Induced”[MeSH] OR “Pre-Eclampsia”[MeSH]))) AND ((“Health Status Disparities”[MeSH] OR “Racism”[MeSH] OR “Ethnicity”[MeSH] OR “Race Factors”[MeSH]))) AND (2020:2025[dp]).

Given the need for a timely synthesis of evidence to support discussions on AI and maternal health, a rapid review methodology was chosen over a traditional systematic review. Although the rapid review maintains rigor in the search and extraction, methodological simplifications were adopted to accelerate the process: Reducing duplication in the title and abstract selection phase may increase the risk of omitting relevant studies (selection bias), and the decision to focus on indexed databases and peer-reviewed literature aims to ensure the technical quality of the AI models discussed, but may introduce publication bias, since negative results or early-stage prototypes (common in technical reports or theses) may not have been captured. Unlike a systematic review, which seeks total exhaustiveness and uses double-checking at all stages to minimize human error, this work prioritizes the identification of the main trends and critical gaps regarding racial bias in AI, recognizing that the speed of the method imposes limits on the absolute generalization of the findings.

The selection of studies occurred in two stages. Initially, titles and abstracts were screened using web Rayyan [16]. The initial screening of titles and abstracts was performed by a single reviewer, with a subsequent review of 20% of the sample by a second senior reviewer to ensure methodological rigor, in accordance with the guidelines for rapid reviews. The selection of studies and data extraction were performed independently by two reviewers. Disagreements were resolved by consensus or by the intervention of a third reviewer. Subsequently, the full texts were evaluated according to the inclusion and exclusion criteria, recording the reasons for exclusion of each study. A flowchart adapted from the PRISMA was developed to document the selection process [17].

Data extraction was performed using a standardized form that captured the following domains: (1) bibliographic information (author, year, country); (2) methodological characteristics (study design, sample size, population characteristics); (3) details of the AI intervention or model (type of algorithm, input variables used, validation procedures, performance metrics); (4) racial and ethnic variables (categories used, proportion of Black or Indigenous women, stratified results, and whether race/ethnicity was included as a predictor or effect modifier); (5) maternal outcomes related to hypertensive disorders or postpartum hemorrhage; (6) reported measures of association or predictive performance stratified by race/ethnicity; and (7) equity-related elements, such as identification of algorithmic bias, data representativeness, or unequal model performance across racial groups. The form also included fields for study limitations, gaps identified, and authors’ recommendations for equity and AI governance.

The presentation of the results included a detailed description of the number of studies identified, included and excluded, distribution by country, racial population, type of AI and outcome, and a selection flowchart. The findings were discussed in the context of racial inequalities, emphasizing studies that reported specific data on Black women, highlighting where knowledge gaps persisted. The limitations of the rapid review were explained, including the restricted search to certain databases, the absence of grey literature, and possible biases arising from screening by a single reviewer, as well as the absence of meta-analysis. To synthesize evidence and represent complex, multivariate data, bibliometric analysis and computational visualization techniques were applied. The geographical distribution of scientific production was generated through spatial mapping to identify global asymmetries and data gaps in the Global South. Network analysis was used to identify the centrality of terms and thematic co-occurrence, allowing visualization of the connectivity between concepts such as “structural racism,” “equity,” and “prediction algorithms.” Additionally, the Sankey diagram was used to map the flow of evidence, integrating the pathologies studied (hypertension and hemorrhage), the AI technologies applied, and the respective outcomes and gaps identified. These tools were fundamental in transforming narrative data into interpretable visual patterns, ensuring that the visualizations function as analytical instruments and not merely illustrative ones.

In this review, the terms “race” and “ethnicity” are used not as biological categories, but as social and political constructs that structure access to resources and the quality of healthcare. In the Brazilian context, the category ‘black women’ encompasses the sum of black and brown women, according to the classification of the Instituto Brasileiro de Geografia e Estatística (IBGE), reflecting how structural racism shapes maternal outcomes. The term “racialized populations” is used to describe groups that, through historical and social processes, have been positioned in a subordinate situation based on phenotypic characteristics or ethnic origins, which directly impacts data collection and the training of AI algorithms.

Since this is a review of published literature, no primary data was collected, therefore approval by an ethics committee was not required. However, ethical criteria were observed regarding respect for copyright, integrity in data presentation, and inclusive and sensitive language concerning racialized populations. Transparency regarding the abbreviated steps and methodological limitations was also ensured, so that readers could adequately assess the reliability and applicability of the findings.

## 3. Results

**Figure 1:**
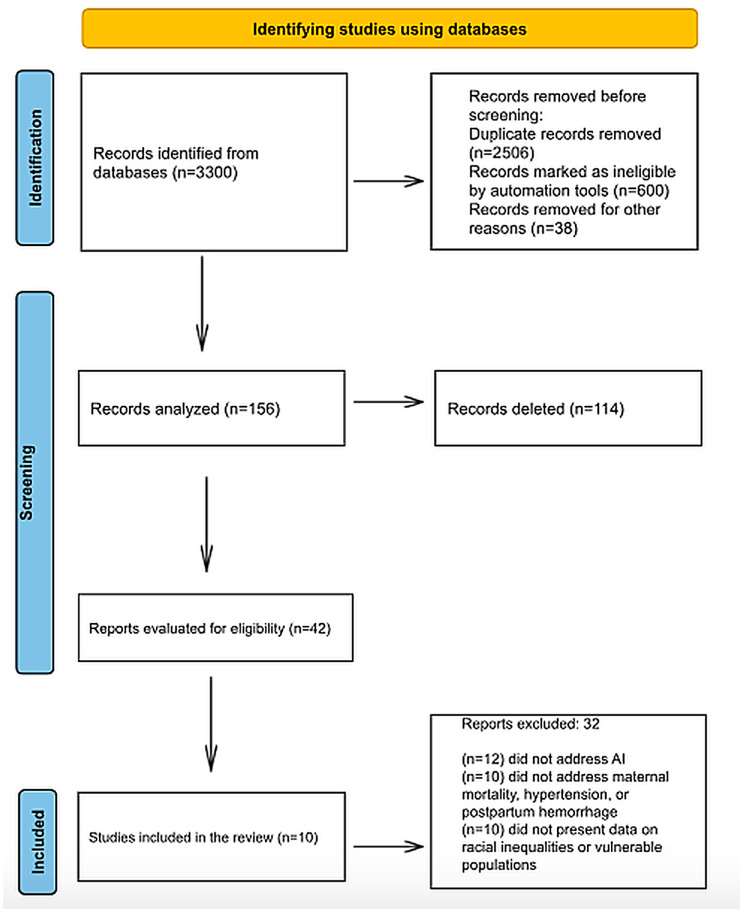
Flowchart of the process for identifying, screening, determining eligibility, and including studies in the review.

Initially, 3300 records were identified in the databases. Before screening, 3144 records were removed: 2506 due to duplication, 600 deemed ineligible by automated tools, and 38 excluded for other reasons. After this stage, 156 records were analyzed, of which 114 were deleted, leaving 42 reports evaluated for eligibility. Of these, 32 were excluded for not meeting the inclusion criteria: 12 did not address artificial intelligence, 10 did not address maternal mortality, hypertension, or postpartum hemorrhage, and 10 did not present data on racial inequalities or vulnerable populations. At the end of the process, 10 studies were included in the review, as shown in Figure 1.

This review included a portion of literature from preprint platforms, a strategy adopted to capture the most recent advances at the intersection of artificial intelligence and maternal health, given the rapid pace of technological innovation in this area. The authors acknowledge that these works have not yet undergone the formal peer review process at the time of this analysis, which implies a lower level of certainty in the evidence and the need for caution in interpreting the technical data. However, these references were selected for their thematic and methodological relevance, serving as indicators of emerging trends.

The results reveal significant disparities in the methodological robustness and scale of the studied populations. Studies conducted in high-income countries, such as those by Jardine et al. and Guan et al., rely on large samples (n > 900,000) and consolidated electronic health record databases, whereas research from low- and middle-income settings presents smaller samples and more fragmented designs, limiting the generalizability of AI models to these contexts. In addition, most studies rely on retrospective validation, with few prospective tests or external audits addressing racial bias, which may compromise the clinical reliability of the reported performance metrics. The literature also shows heterogeneity in how race and ethnicity are operationalized. North American studies generally use self-reported racial categories aligned with census classifications, while Brazilian research tends to treat race as a marker of social vulnerability and structural inequality. The review examined whether AI models considered race as an isolated demographic variable or incorporated associated social determinants, avoiding the risk of interpreting algorithmic disparities as biological differences (Supplementary Materials).

To synthesize the evidence, results were presented through narrative description and visual tools, including tables, charts, and figures. A descriptive table summarized key information from the included studies—author, year, country, design, intervention or exposure, and main findings—providing an overview of the evidence on the use of AI in predicting maternal complications related to hypertension and postpartum hemorrhage. The geographical distribution of the ten included studies shows a predominance of research conducted in the United States (n = 4) and the United Kingdom (n = 2), followed by Iran (n = 1), Kenya (n = 1), and two multicenter/global studies (n = 2). This distribution highlights the concentration of research and data infrastructure in high-income countries, particularly in North America and Europe, and the scarcity of studies in lower-income settings, especially in the Global South.

Overall, the reviewed studies demonstrate that AI and machine learning tools are increasingly used to predict severe obstetric complications, particularly postpartum hemorrhage and hypertensive disorders of pregnancy, often showing high predictive performance. However, recent literature emphasizes that equitable implementation requires diverse and representative datasets, validation in racially diverse populations, and explicit attention to algorithmic bias. While AI shows considerable potential to support the prevention of adverse maternal outcomes, its equitable effectiveness depends on the inclusion of vulnerable populations and on predictive models that incorporate race, socioeconomic conditions, and contextual determinants of care, as shown in Table 1.

The ten studies included in this rapid review collectively demonstrate the increasing application of AI and machine learning (ML) in predicting critical maternal complications, particularly PPH and hypertensive disorders of pregnancy. Prospective, retrospective, cohort, and model development studies across diverse geographical contexts including the USA, Iran, Kenya and multicenter settings consistently showed high predictive performance [25,27]. These findings indicate that AI has substantial potential to identify women at elevated risk of severe maternal outcomes, enabling timely interventions and potentially reducing mortality. Several studies highlighted that black women and other racialized populations experience higher rates of maternal mortality due to PPH and hypertensive disorders, and that AI models may perpetuate existing disparities if racial diversity is not adequately represented in the training datasets [21,23,26]. Only a few studies explicitly incorporated racial variables or analyzed differential performance across racial groups, demonstrating a critical gap in the equitable application of AI in maternal health. Reviews and methodological papers further emphasize that the inclusion of diverse populations and socio-demographic variables is essential to prevent bias and ensure AI-driven tools contribute to reducing, rather than exacerbating, maternal health disparities.

Table 2 highlights the main methodological and structural gaps in the analyzed studies, emphasizing the absence of external validation, the use of non-representative databases, the scarcity of racial variables, the low transparency of the algorithms, and the limitations of technological infrastructure in low-income countries. Furthermore, it shows that most AI models focus on predicting intermediate obstetric complications, such as postpartum hemorrhage and pre-eclampsia, without directly assessing the impact on maternal mortality, which restricts the applicability of the results in real-world contexts.

Table 3, in turn, presents a set of practical and ethical recommendations to promote the equitable implementation of these technologies. Among the proposed measures are strengthening data diversity, racial and contextual validation of models, transparency and auditability of algorithms, clinical training of professionals, integration of AI tools with public policies on racial equity, and the promotion of interdisciplinary research.

Taken together, the two frameworks indicate that the technical advancement of AI in reproductive health must be accompanied by an agenda of reproductive justice, diversity, and ethical governance, in order to ensure that technological innovation effectively contributes to reducing, rather than perpetuating, racial inequalities in maternal mortality. Finally, the results were organized according to previously identified thematic categories: AI technical performance, racial equity and validation, implications for practice, and limitations and future directions, which allowed for a structured discussion aligned with the objective of the review.

## 4. Discussion

The studies included in this review show that AI and ML models perform well in predicting serious maternal complications, especially PPH and hypertensive disorders of pregnancy, often exceeding 0.95 in high-income cohorts. For example, in a large retrospective study in the United States with 30,867 women, the ML model achieved an AUROC of 0.979 for PPH prediction. This level of performance confirms the potential of AI to anticipate high-risk events, which are known to be responsible for the majority of preventable maternal deaths [28,29,30]. However, recent systematic reviews indicate that most models remain internally validated, with a scarcity of external validation and execution in middle- or low-income contexts, which limits generalization [31].

Regarding the results directly observed in the studies included in this review, empirical evidence indicates that AI models have high predictive accuracy for postpartum hemorrhage and pre-eclampsia, but lack robust validation in racial subgroups. For example, Jardine et al. empirically demonstrated, in a cohort of nearly one million births, that the risk of hemorrhage is directly associated with ethnicity, and that models incorporating these variables tend to be more accurate for diverse populations [7]. Similarly, Guan et al. observed real disparities in the escalation of care and transfusion rate for postpartum hemorrhage in Black women, suggesting that AI training data already carry historical care biases that may compromise future prediction [8].

On the other hand, the analysis of equity in AI must integrate normative and ethical considerations drawn from the specialized literature on social justice. The lens of Critical Race Theory, that technology is not neutral, but a social construct that can reproduce and automate existing power structures [22]. In this sense, ethics in AI requires that algorithmic justice (fairness) be understood as a political commitment to equity, and not merely as a technical adjustment of statistical weights. Lima & Gaudenzi reinforce that institutional racism shapes subjectivities in healthcare; therefore, normatively, an ethical AI system must be designed to proactively mitigate these human flaws [23].

Even more serious is the fact that few studies have presented specific analysis by race or have included racial variables in a robust way, which constitutes a critical gap when considering the known disparities: black women in the US often have maternal mortality rates 2–4 times higher than white women [32].

Among the reviewed articles, only a few mentioned the inclusion of race as a variable or the need for validation in racialized populations, such as the study on pre-eclampsia in the United Kingdom, which found that the inclusion of racial variables improved the model’s accuracy [33]. However, this type of inclusion remains the exception, which reinforces the risk that AI may reproduce or even amplify existing inequalities if applied without attention to the representativeness of the data. When models are trained predominantly on white women, they learn patterns specific to white women’s physiology, presentation of disease, and healthcare access. For example, if vital signs are monitored more frequently in white women, the model learns to rely on frequent vital sign measurements. When applied to Black women with less frequent monitoring, the model may fail because it receives ‘incomplete data.’ This is not a biological difference, but a difference in data quality that reflects healthcare inequities. Review studies highlight that algorithmic bias can occur when racialized groups are underrepresented or when models are trained on predominantly white or high-income populations [21,34].

In terms of a global context, the findings of studies in low- and middle-income countries (e.g., the Kenya study on PPH) are particularly relevant: they demonstrate that AI models can work in vulnerable and resource-poor environments, suggesting applicability beyond the centers of excellence in high-income countries [24]. However, even in these contexts, there is a lack of data that allows for the segmentation of vulnerable racial/ethnic groups, which implies that the benefits of AI may not reach Black or Indigenous women equitably. A recent international review emphasizes that, for HPP prediction, there is great heterogeneity among studies, many with limited validation and virtually none focused on racialized populations or specific regions of vulnerability [31].

From the perspective of maternal mortality due to gestational hypertension, the use of AI still shows less evidence compared to PPH, but presents promising indications, for example, the Pre-eclampsia Integrated Estimate of Risk—Machine Learning (PIERS-ML) model for pre-eclampsia improved the identification of women at high risk of adverse outcome within 2 days [35].

The discussion on algorithmic fairness is central: well-designed AI tools can be powerful allies in reducing inequalities, but if implemented without attention to the diversity of the database, validation in vulnerable groups, and algorithm transparency, they can perpetuate the status quo. Studies such as Fair Machine Learning for Healthcare demonstrate that racial, socioeconomic index, and intersectional factors (e.g., race + sex + class) interact in the performance and impact of AI models, so algorithmic justice requires explicit recognition of these dimensions [36].

In maternal health, it is essential to distinguish between algorithmic bias and structural inequalities. Consider two scenarios in predicting postpartum hemorrhage (PPH): Algorithmic Bias: A model trained on predominantly white women learns that “elevated blood pressure” predicts PPH. However, Black women receive fewer prenatal visits and less frequent blood pressure monitoring due to systemic racism. When applied to Black women, the model underestimates risk because it receives incomplete data not because of biological differences, but because of healthcare inequities. This is algorithmic bias: the model produces worse predictions for Black women. Reflecting Structural Inequality: A model learns that “living in a neighborhood with limited obstetric services” predicts adverse outcomes. Black women are overrepresented in such neighborhoods due to segregation and disinvestment. When the model predicts a higher risk for Black women, it is not biased; it is accurately reflecting a real structural inequality. The problem is not the model, but the underlying inequality. The distinction matters: if a model exhibits algorithmic bias, we must improve the model. If a model reflects structural inequalities, improving the model alone is insufficient; we must address underlying causes. However, the reviewed studies do not make this distinction explicit, and most did not report performance stratified by race. This represents a critical gap for equitable implementation [37].

The reviewed studies fail to address intersectionality and how multiple, overlapping forms of discrimination affect maternal health [38]. Black women experiencing maternal mortality are not a homogeneous group: they include low-income women, rural women, women experiencing intimate partner violence, women with substance use disorders, and women navigating immigration status [39]. These intersecting vulnerabilities interact in ways that single-axis analysis cannot capture. An AI model trained on data from women with regular prenatal care may fail for women experiencing homelessness or violence, populations that are disproportionately represented among Black women who die during pregnancy or childbirth. A model reporting 95% accuracy “for Black women” may mask 70% accuracy for low-income Black women in rural areas. This represents a hierarchy of vulnerability where the most vulnerable are left behind [40]. To ensure equity, AI systems must: (1) evaluate performance for intersectional subgroups; (2) involve women with lived experience of multiple marginalization; and (3) recognize that technology alone cannot address structural inequities driving maternal mortality [41,42].

A cross-sectional limitation of the reviewed studies relates to the outcome of maternal mortality itself; many focused on predicting risk or serious complications (HPP, pre-eclampsia) as a proxy for mortality, but few explicitly assessed maternal deaths or included mortality stratified by race. This compromises the certainty of inferring that a reduction in mortality among Black women will be achieved with the adoption of AI. Recent reviews indicate that for PPH, the overall mortality rate remains high in low-income countries, and that most AI models have not yet been tested in contexts where mortality is higher, a context where Black and Indigenous women are underrepresented [29].

The included studies demonstrate that AI and machine learning exhibit high technical performance in predicting serious obstetric complications, especially postpartum hemorrhage and hypertensive disorders of pregnancy [27]. Other investigations, such as those by Susanu et al. (2024) and Ahmadzia et al. (2024), have demonstrated similar accuracy in predicting hemorrhagic risk, with the ability to identify high-risk women early [18,19]. Recent systematic reviews also indicate that these models outperform conventional methods and can reduce critical delays in obstetric emergency settings [28]. However, most of the evidence comes from high-income settings, with limited internal validation and a scarcity of multicenter external tests, which compromises the generalizability of the findings [20,25].

Racial equity in model validation is one of the main gaps identified. Only a small number of studies have included racial variables or evaluated the performance of algorithms among different ethnic groups [26]. The work of Ansbacher-Feldman et al. (2022) showed that the inclusion of racial characteristics and biomarkers increased the accuracy in predicting pre-eclampsia, but this approach is still the exception [26]. Narrative research and methodological reviews, such as those by McAdams & Green (2024) and Mapari et al. (2024), warn that models based on predominantly white databases may reproduce or amplify existing racial disparities in maternal mortality [20,21]. Furthermore, the multicenter study by Mehrnoush et al. (2023) in a Kenyan population showed that, although AI can work in resource-limited contexts, the absence of racial stratification and diversity data compromises its equitable effectiveness [24]. Thus, external validation in racialized populations is an essential step to ensure algorithmic justice and representativeness in maternal health [22,23].

The practical implications of integrating AI into maternal health are broad. Effective predictive models can optimize care flows, identify at-risk pregnant women early, and support clinical decisions based on real-time data [18,19,27]. The responsible adoption of these tools requires staff training, interoperability with electronic records, and continuous supervision to avoid biased interpretations. Recent literature argues that AI systems should be evaluated from an equity perspective, including sensitivity analyses stratified by race/ethnicity and socioeconomic conditions [21,23]. In highly vulnerable contexts, such as in low- and middle-income countries, the ethical use of AI can expand access to early detection of complications, provided it is associated with policies to strengthen data infrastructure and governance [24,25]. Therefore, AI has the potential to reduce inequalities if implemented in an inclusive, transparent, and supervised manner.

Analysis of the selected studies reveals a focus on predicting events such as postpartum hemorrhage and pre-eclampsia, which are proximal causes but not the ultimate outcome of maternal mortality. It is crucial to question whether the accuracy achieved in these models translates into an effective reduction in deaths in resource-limited settings. Addressing racial inequities requires more than accurate diagnoses; it demands a transformation in how healthcare ‘sees, says, and does’. In healthcare systems with precarious infrastructure, the predictive capacity of AI may be underutilized if there are no resources for immediate interventions following the algorithmic alert [43,44,45,46,47].

Maternal mortality is profoundly influenced by structural racism, which dictates disparities in access to and quality of prenatal care. The application of Critical Race Theory suggests that focusing only on intermediate outcomes may mask the fact that Black women die not only from the pathology itself, but also from institutional negligence and geographical and socioeconomic barriers. Therefore, the high accuracy of a model for pre-eclampsia does not guarantee survival if the health system does not consider the social determinants that prevent this racialized woman from reaching the referral center in a timely manner [25,47,48,49].

Furthermore, the transposition of AI models developed in high-income countries to the Brazilian reality should be viewed with caution. Technology can act as an instrument for perpetuating white privilege if its design ignores the specificities of marginalized populations. In environments with budgetary constraints, investing in predictive software without correcting human racial bias in clinical decisions can result in technological optimization that does not reach those who need it most, keeping direct maternal mortality rates stagnant in the black population [25].

Although Early Warning Systems (EWS) are based on vital signs considered objective physiological measures, their clinical effectiveness is profoundly influenced by disparities in monitoring and the quality of the data entered. Evidence suggests that the frequency of vital sign recordings and the completeness of data in electronic health records can vary according to the patient’s ethnic-racial profile, a phenomenon often derived from implicit biases in the nursing and medical staff. When an EWS algorithm operates on incomplete data or data collected less frequently in Black women, its ability to detect early clinical deterioration is severely impaired, reducing the system’s reliability precisely for the highest-risk groups [50,51].

In addition to monitoring failures, the standardization of baseline thresholds in AI models often ignores physiological variations associated with social determinants of health. Studies indicate that algorithms trained on predominantly Caucasian populations may not accurately reflect the hemodynamic profiles of racialized populations, which may exhibit variations in blood pressure regulation and response to oxidative stress due to chronic structural racism. If early warning systems are not calibrated to recognize these nuances and instead apply unfounded biological racial corrections, there is a risk of underestimating the severity of conditions such as pre-eclampsia in women from ethnic minorities [52,53].

The true usefulness of an early warning system (AWS) is defined by the clinical response that the alert triggers. Research demonstrates that there is a significant disparity in the speed and aggressiveness of care provided after a system indicates clinical risk; Black women are less likely to receive timely interventions, such as the rapid administration of antihypertensives or hemorrhage protocols, even when the system alert is identical to that of a white patient. Therefore, confidence in early warning technology must be accompanied by equity protocols that ensure that the physiological signal captured by AI results in fair and standardized clinical action for all ethnicities [54,55].

Analysis of AI models for predicting serious obstetric complications reveals that dynamic hemodynamic variables are the most robust predictors for classification performance. Recent studies indicate that systolic and diastolic blood pressure, especially when analyzed in time series rather than as isolated measurements, constitute the most significant characteristics in machine learning algorithms for predicting pre-eclampsia. In addition, heart rate variability has emerged as an early sign of autonomic instability, allowing deep learning models to anticipate hypertensive crises before traditional clinical thresholds are reached, increasing the window of opportunity for intervention [56,57].

In the context of postpartum hemorrhage, the integration of conventional vital signs with real-time laboratory markers significantly increases the accuracy of alert systems. In addition to heart rate and oxygen saturation, which show late changes in volume compensation, markers such as serum lactate and the shock index (ratio between heart rate and systolic blood pressure) have been identified as highly informative variables for predicting the need for massive transfusion. The interpretability of these models reveals that the combination of an abrupt drop in peripheral saturation with a sustained increase in the shock index offers the highest positive predictive value for identifying occult hypovolemic shock [58,59].

The generalization of these models, however, depends on the stability of these variables in different population profiles. Although temperature and respiratory rate contribute to the detection of maternal sepsis, their sensitivity for hypertension and hemorrhage is lower when compared to laboratory data on renal (creatinine) and hepatic (transaminases) function. Current literature emphasizes that the inclusion of specific biomarkers, such as the sFlt-1/PlGF ratio in hybrid models, not only improves technical performance (AUROC) but also increases clinical relevance by aligning algorithmic prediction with the pathophysiology of pre-eclampsia, allowing for more precise risk stratification in highly complex settings [60,61].

It is imperative to distinguish between data-driven bias and the structural inequalities that shape the healthcare ecosystem. The algorithmic bias observed in the reviewed studies stems predominantly from the underrepresentation of racialized populations in training databases, resulting in models with lower predictive accuracy for Black women. However, structural inequality precedes the data; it manifests itself in disparities in access to diagnostic tests and the quality of care received, generating already biased clinical records. Therefore, AI not only inherits these statistical errors but also risks encoding historical socioeconomic and racial disparities as if they were biological truths, transforming social inequities into risky mathematical models [51,62].

Regarding the level of readiness for clinical application, most of the AI tools analyzed are still in early stages of Technology Readiness Levels (TRL), focused on internal and retrospective validation. Significant practical barriers to implementation exist, such as a lack of interoperability between electronic health record systems and the absence of computing infrastructure in resource-limited healthcare facilities. Furthermore, the regulatory landscape for AI-based medical devices in obstetrics remains fragmented. While health agencies have made progress in guidelines for decision support algorithms, specific regulations requiring mandatory racial equity audits before commercial approval of these systems are still lacking [63,64].

The transition from the research bench to the hospital bedside requires models to demonstrate not only high technical performance but also clinical interpretability and legal soundness. The generalization of these models is limited by a scarcity of external validation in “real-world” contexts, where human operator bias and structural flaws in the healthcare system can negate the predictive benefits of the technology. Thus, the readiness for the clinical use of AI in maternal health depends on ethical governance that integrates constant algorithmic surveillance and proactive monitoring of racial outcomes after implementation [65,66].

The limitations observed in this review reflect the current stage of AI research in maternal mortality. Most studies assessed intermediate outcomes (PPH, pre-eclampsia) rather than maternal mortality itself, which limits the ability to infer the true impact of AI tools on preventing deaths [19,25]. An important limitation of the evidence identified in this review is that most studies focused on intermediate obstetric outcomes, such as postpartum hemorrhage and hypertensive disorders of pregnancy, rather than maternal mortality itself. Although these conditions are major contributors to maternal deaths worldwide, predictive models developed for these complications cannot be interpreted as direct predictors of maternal mortality. Therefore, the conclusions of this review should be interpreted with caution, as the current evidence primarily reflects the capacity of artificial intelligence models to identify risks for severe maternal complications rather than maternal death per se.

A major constraint was the scarcity of consistent racial and ethnic data: because most studies did not report performance stratified by race, this review was unable to compare algorithmic behavior across groups, which is central to equity analysis. Likewise, the absence of external validation, subgroup analyses, and cost-effectiveness assessments reduces the certainty of the available evidence. The limitations of this study reflect the cumulative impact of methodological constraints inherent in the rapid review format. Single-reviewer screening and the exclusion of grey literature may have resulted in the omission of AI models in the prototyping stage or technical reports discussing implementation failures in local contexts. Furthermore, the absence of a formal and exhaustive assessment of the risk of bias limits the ability to grade the certainty of the synthesized evidence. These limitations, coupled with the heterogeneity of the included study designs and the inconsistency in reporting the race/ethnicity variable, imply that the findings should be interpreted as emerging trends rather than definitive evidence. The lack of external validation in most of the reviewed models prevents the generalization of these results to healthcare systems outside the high-income centers where they were developed.

Future directions include: (1) developing multicenter and racially diverse datasets that explicitly include Black and Indigenous women; (2) conducting external validation in low-resource settings; (3) ensuring algorithmic transparency and reporting of performance metrics by subgroup; and (4) strengthening ethical and regulatory frameworks for equitable AI development in reproductive health [20,23,26,27]. Finally, interdisciplinary approaches integrating data science, epidemiology, and racial studies are essential for AI to fulfill its promise of reducing rather than perpetuating global maternal inequalities.

## 5. Conclusions

This review demonstrates that artificial intelligence models show promising technical performance in predicting severe obstetric complications, particularly hypertensive disorders of pregnancy and postpartum hemorrhage. However, it is important to acknowledge that most of the studies included in this review focused on these intermediate maternal outcomes rather than maternal mortality itself. Although these complications are major contributors to maternal death, the current evidence primarily reflects the ability of AI tools to predict risks for severe maternal morbidity rather than maternal mortality directly. Furthermore, the clinical applicability of these technologies in racially diverse and socially vulnerable populations remains insufficiently validated. Evidence suggests that algorithmic bias may persist when predictive models are developed using unrepresentative datasets, potentially reinforcing existing health inequities. For this reason, caution is warranted in the immediate implementation of these systems without prior evaluation of their performance across different racial and socioeconomic groups.

Future research should prioritize the development of more diverse and representative databases, algorithmic transparency, and the incorporation of social determinants of health into predictive models. In addition, external validation studies and real-world implementation research are necessary to assess the clinical impact of AI-based tools in maternal health care. Ultimately, the implementation of artificial intelligence should be understood as a complementary strategy within broader efforts to strengthen obstetric care and address structural determinants of maternal health. Aligning technological innovation with reproductive justice and racial equity agendas will be essential to ensure that AI contributes to reducing preventable maternal complications and, potentially, maternal mortality among historically marginalized populations.

## Figures and Tables

**Table 1:** Applications of artificial intelligence in predicting maternal mortality: summary of included studies.

Author/Year	Country	Study Design	Intervention/Exposure	Key Findings
Susanu et al., 2024 [18]	Multicenter	Prospective study	ML algorithms for predicting intra- and postpartum hemorrhage	Models predicted hemorrhage risk with high accuracy; need for validation in racialized populations to reduce mortality in vulnerable groups
Ahmadzia et al., 2024 [19]	USA	Model development study	ML for predicting postpartum hemorrhage and transfusion	ML identified high risk of postpartum hemorrhage; highlights need for testing in black women
Mapari et al., 2024 [20]	Global	Narrative review	AI in maternal health	AI can improve early detection; racial disparities may persist if black women underrepresented
McAdams & Green, 2024 [21]	USA	Narrative review	AI in obstetrics, maternal-fetal medicine, and neonatology	AI tools may reproduce biases, increasing mortality in Black women; emphasize hypertension and hemorrhage
Asiedu et al., 2024 [22]	UK	Dataset development	OxMat multimodal dataset for maternal-infant health AI	Robust dataset; potential to mitigate disparities including mortality from hemorrhage or hypertension; validation in black women needed
Liu et al., 2023 [23]	USA	Annotation tool development	AI tool for analyzing maternal safety reports	Identified disparities in risk factors; AI may help mitigate higher postpartum hemorrhage mortality in black women
Mehrnoush et al., 2023 [24]	Ira	Observational study	ML for predicting postpartum hemorrhage	Models predicted hemorrhage in vulnerable populations; populations with limited care access show higher maternal mortality
Shah et al., 2023 [25]	Kenya	Retrospective study	ML for predicting postpartum hemorrhage	XGBoost predicted hemorrhage; socio-economic factors, similar to those affecting black women, influence mortality
Ansbacher-Feldman et al., 2022 [26]	UK	Cohort study	ML for predicting preeclampsia using first-trimester data	Inclusion of racial variables improved accuracy; black women have higher hypertensive disorder risk
Westcott et al., 2022 [27]	USA	Retrospective cohort	ML in 30,867 women	Models predicted hemorrhage; racialized populations, especially black women, have higher mortality; need race-specific validation

**Table 2:** Main gaps identified in studies on artificial intelligence and maternal mortality.

Category	Description of the Gap	Examples of Impact or Evidence
**High technical performance without external validation**	Most AI and ML models showed high accuracy (AUROC > 0.90), yet remained internally validated without testing in other populations or settings	Studies from the USA and Iran confirmed high predictive accuracy for postpartum hemorrhage and hypertensive disorders, but lacked multicenter replication (Susanu et al., 2024 [18]; Ahmadzia et al., 2024 [19]; Shah et al., 2023 [25]; Westcott et al., 2022 [27])
**Lack of racial or ethnic variables**	Only a small number of studies incorporated race or ethnicity in model development, limiting assessment of algorithmic fairness	Few models stratified performance by race, with rare examples of inclusion improving accuracy (Ansbacher-Feldman et al., 2022 [26]; McAdams & Green, 2024 [21]
**Non-representative databases**	Most datasets originated from high-income countries, with limited participation of Black, Indigenous, or low-income women	Models developed in the USA and UK predominantly reflected high-resource clinical settings (Ahmadzia et al., 2024 [19]; Liu et al., 2023 [23]; Asiedu et al., 2024 [22])
**Low transparency and ethical governance**	Limited disclosure of algorithm structure, selection criteria, or data-handling procedures reduced reproducibility	Narrative reviews highlighted the absence of open-source models and insufficient ethical oversight (Mapari et al., 2024 [20]; McAdams & Green, 2024 [21])
**Technological infrastructure inequalities**	Few studies addressed challenges of implementing AI in resource-limited or low-connectivity contexts.	The study conducted in Kenya demonstrated feasibility but emphasized structural barriers (Mehrnoush et al., 2023 [24])
**Focus on intermediate outcomes**	Most models predicted severe complications such as PPH or pre-eclampsia rather than maternal deaths directly	Predictive models were used as proxies for mortality, limiting conclusions on life-saving effectiveness (Susanu et al., 2024 [18]; Westcott et al., 2022 [27])

**Table 3:** Recommendations for equitable implementation of IA in reproductive health.

Axis of Action	Specific Recommendations	Rationale and Supporting Evidence
**Data diversity**	Establish multicenter and racially diverse datasets including black, Indigenous, and low-income women	Heterogeneity in datasets is essential for fair model performance (Asiedu et al., 2024 [22]; Ansbacher-Feldman et al., 2022 [26])
**Racial and contextual validation**	Conduct external validation in racially and socioeconomically diverse populations	Lack of validation across racial subgroups was consistently identified as a major gap (Liu et al., 2023 [23]; Westcott et al., 2022 [27])
**Transparency and auditability**	Disclose model architecture, input variables, and subgroup performance metrics	Reviews emphasize the need for transparency and algorithmic accountability (Mapari et al., 2024 [20]; McAdams & Green, 2024 [21])
**Clinical training and supervision**	Train healthcare professionals to interpret AI outputs critically and ensure human oversight	Narrative studies recommend clinician education to prevent overreliance on automated tools (McAdams & Green, 2024 [21]; Liu et al., 2023 [23])
**Integration with public policies**	Align AI-based interventions with national strategies for racial equity and maternal health	Implementation should occur within ethical and policy frameworks to avoid widening inequalities (Asiedu et al., 2024 [22]; Mehrnoush et al., 2023 [24])
**Interdisciplinary research**	Promote collaboration among data scientists, clinicians, and social scientists focused on racial equity	Interdisciplinary approaches strengthen contextual understanding and ethical use of AI (Mapari et al., 2024 [20]; McAdams & Green, 2024 [21])

## Data Availability

The data presented in this study are available in Open Science Framework (OSF) at https://doi.org/10.17605/OSF.IO/B76MS.

## Supplementary Materials

The following supporting information can be downloaded at: https://www.mdpi.com/article/10.3390/epidemiologia7030081/s1, Figure S1: Geographic distribution included studies on AI in maternal mortality prevention due to hypertensive disorders and postpartum hemorrhage, highlighting study concentration in high-income countries; Figure S2: Network of countries and research themes in AI and maternal mortality; Figure S3: Sankey Diagram of AI types, maternal complications and regions.

## Abbreviations

AI	Artificial intelligence
EMBASE	Excerpta Medica dataBASE
EWS	Early warning systems
LILACS	Literatura Latino-Americana e do Caribe em Ciências da Saúde
MEDLINE	Medical Literature Analysis and Retrieval System Online
ML	Machine learning
OSF	Open Science Framework
PICO	Population, exposure/intervention, comparator and outcomes
PIERS-ML	Pre-eclampsia Integrated Estimate of Risk-Machine Learning
PPH	Postpartum hemorrhage
PRISMA	Preferred Reporting Items for Systematic Reviews and Meta-Analyses
PubMed	U.S. National Library of Medicine
