# Supplementary material for: Racial Disparities and the Use of Artificial Intelligence for Predicting Maternal Mortality: A Literature Review
Source: Epidemiologia (Basel). 2026 Jun 10;7(3):81. doi: 10.3390/epidemiologia7030081 (PMC13297638; doi:10.3390/epidemiologia7030081)
Supplement: Supplementary file 1 [file epidemiologia-07-00081-s001.zip › epidemiologia-4051643-supplementary.pdf]

Figure S1. Geographic distribution included studies on AI in maternal mortality prevention due to hypertensive disorders and postpartum hemorrhage, highlighting study concentration in high-income countries

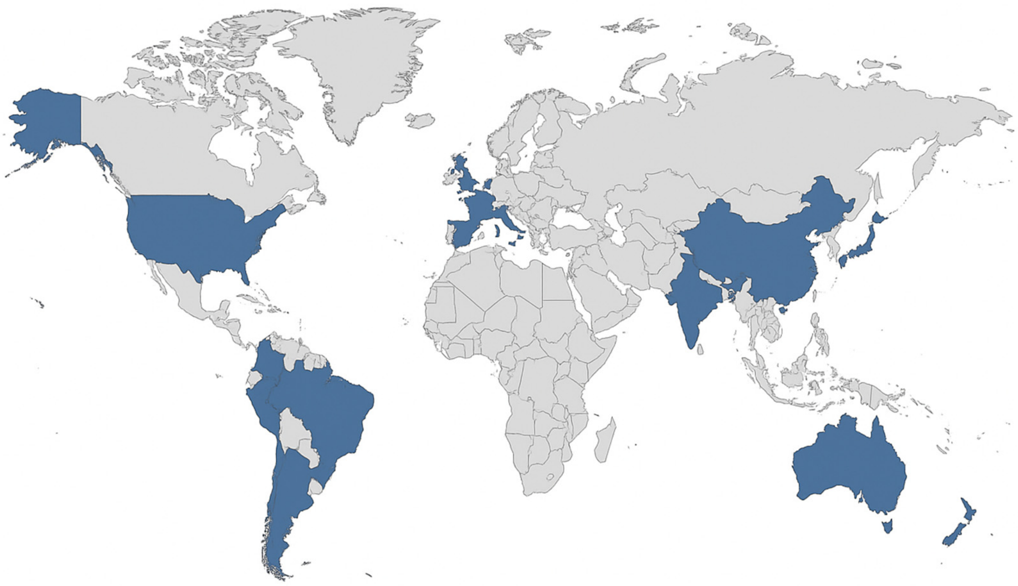

To support the spatial analysis of scientific production, a thematic world map was developed, representing the geographical distribution of the included studies by country and continent. The map was designed in a minimalist style, highlighting the countries where the research was conducted (United States, United Kingdom, Iran, Kenya, and global multicenter studies), allowing visualization of regional asymmetries in scientific production on the topic, as illustrated in Figure 2.

Figure S2. Network of countries and research themes in AI and maternal mortality

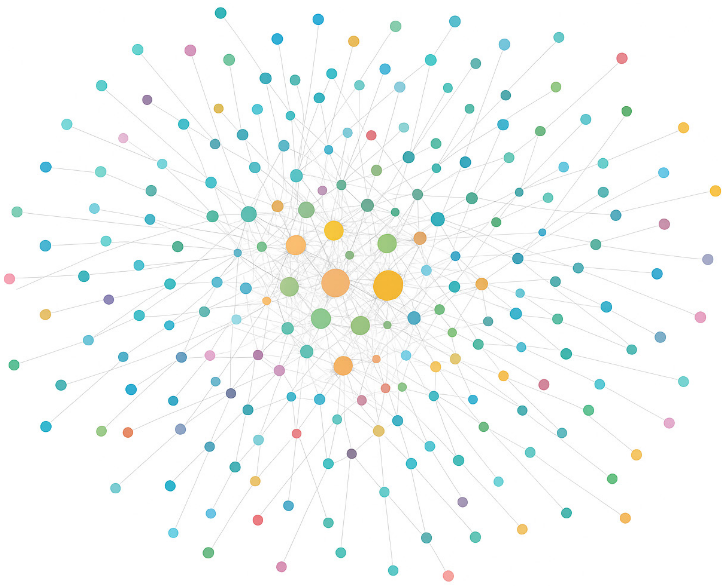

This network visualization represents connections between countries (United States, United Kingdom, Iran, Kenya, Global) and dominant research themes (postpartum hemorrhage, hypertensive disorders, equity, bias, AI validation) identified in the literature. Node size reflects the frequency of occurrence, while link density represents thematic co-occurrence across studies. The structure highlights strong clustering around AI bias and equity in high-income countries and sparse connections in lower-income contexts, underscoring global disparities in research production.

Figure S3. Sankey Diagram of AI types, maternal complications and regions

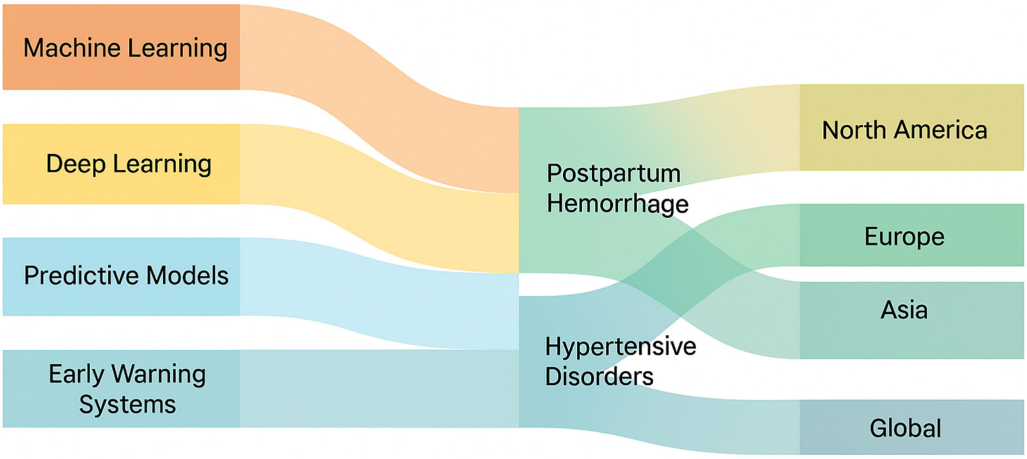

The Sankey diagram visualizes the relationship between the types of artificial intelligence used (machine learning, deep learning, predictive models, early warning systems), maternal complications (postpartum hemorrhage and hypertensive disorders of pregnancy), and the regions where studies were conducted. The width of the flows represents the relative frequency of studies across these dimensions, revealing that most AI applications focused on postpartum hemorrhage and were concentrated in North America and Europe.
